# Retraction: Hong Liu, Shi‐Ying Ren, Yan Qu, Cui Liu, Yi Zhang, Xiang Qing Li, Hong Ma. MiR‐194‐5p inhibited metastasis and EMT of nephroblastoma cells through targeting Crk. The Kaohsiung Journal of Medical Sciences, Volume 36, Issue 4 Apr 2020. Pages 265–273. https://doi.org/10.1002/kjm2.12180

**DOI:** 10.1002/kjm2.12727

**Published:** 2023-06-28

## Abstract

The above article, published online on 31 December 2019, in Wiley Online Library (wileyonlinelibrary.com), has been retracted by agreement between Hong Ma, Cui Liu and Yan Qu, the journal Editor‐in‐Chief, Wan‐Long Chuang, and John Wiley and Sons Ltd. The retraction has been agreed because the article was submitted and approved for publication by Hong Liu without consent from the named co‐authors Shi‐Ying Ren, Yan Qu, Cui Liu, Yi Zhang, Xiang Qing Li, and Hong Ma. Other authors were not available for a final confirmation of the retraction.

The above article, published online on 31 December 2019, in Wiley Online Library (wileyonlinelibrary.com), has been retracted by agreement between Hong Ma, Cui Liu and Yan Qu, the journal Editor‐in‐Chief, Wan‐Long Chuang, and John Wiley and Sons Ltd. The retraction has been agreed because the article was submitted and approved for publication by Hong Liu without consent from the named co‐authors Shi‐Ying Ren, Yan Qu, Cui Liu, Yi Zhang, Xiang Qing Li, and Hong Ma. Other authors were not available for a final confirmation of the retraction.

